# Gastric Cancer Cell Glycosylation as a Modulator of the ErbB2 Oncogenic Receptor

**DOI:** 10.3390/ijms18112262

**Published:** 2017-10-28

**Authors:** Henrique O. Duarte, Meritxell Balmaña, Stefan Mereiter, Hugo Osório, Joana Gomes, Celso A. Reis

**Affiliations:** 1 Instituto de Investigação e Inovação em Saúde, Universidade do Porto, 4200-135 Porto, Portugal; hduarte@ipatimup.pt (H.O.D.); mbalmana@ipatimup.pt (M.B.); smereiter@ipatimup.pt (S.M.); hosorio@i3s.up.pt (H.O.); 2 Institute of Molecular Pathology and Immunology, University of Porto, 4200-135 Porto, Portugal; 3 Institute of Biomedical Sciences Abel Salazar, University of Porto, 4050-313 Porto, Portugal; 4 Faculty of Medicine, University of Porto, 4200-319 Porto, Portugal

**Keywords:** human epidermal growth factor receptor 2 (ErbB2), gastric cancer (GC), glycosylation, sialyl Lewis a (SLe^a^), CA 19.9

## Abstract

Aberrant expression and hyperactivation of the human epidermal growth factor receptor 2 (ErbB2) constitute crucial molecular events underpinning gastric neoplastic transformation. Despite ErbB2 extracellular domain being a well-known target for glycosylation, its glycosylation profile and the molecular mechanisms through which it actively tunes tumorigenesis in gastric cancer (GC) cells remain elusive. We aimed at disclosing relevant ErbB2 glycan signatures and their functional impact on receptor’s biology in GC cells. The transcriptomic profile of cancer-relevant glycosylation enzymes, and the expression and activation of the ErbB receptors were characterized in four GC cell lines. Cellular- and receptor-specific glycan profiling of ErbB2-overexpressing NCI-N87 cells unveiled a heterogeneous glycosylation pattern harboring the tumor-associated sialyl Lewis a (SLe^a^) antigen. The expression of SLe^a^ and key enzymes integrating its biosynthetic pathway were strongly upregulated in this GC cell line. An association between the expression of *ERBB2* and *FUT3*, a central gene in SLe^a^ biosynthesis, was disclosed in GC patients, further highlighting the crosstalk between ErbB2 and SLe^a^ expression. Moreover, cellular deglycosylation and CA 19.9 antibody-mediated blocking of SLe^a^ drastically altered ErbB2 expression and activation in NCI-N87 cells. Altogether, NCI-N87 cell line constitutes an appealing in vitro model to address glycan-mediated regulation of ErbB2 in GC.

## 1. Introduction

Gastric cancer (GC) represents the fifth most incident human malignancy and the third leading cause of cancer-related deaths worldwide [1]. Being frequently diagnosed at advanced stages, at which surgical resection and conventional chemotherapeutic approaches fall short in improving patients’ dismal prognosis, GC remains a globally heavy health burden [2].

Overexpression of the human epidermal growth factor receptor 2 (ErbB2), a direct consequence of the *ERBB2* gene amplification, constitutes a well-established molecular hallmark of multiple human solid tumors, and has been consistently reported as a key molecular driver of gastric carcinogenesis, by triggering the aberrant activation of complex downstream signaling pathways that ultimately dictate malignant cell behavior (reviewed in [3]). Indeed, ErbB2 overexpression is observed in 7% to 34% of GC patients and constitutes a predictive factor of poor disease prognosis [4]. The 185-kDa ErbB2 transmembrane protein belongs to the ErbB epidermal growth factor receptor family of receptor tyrosine kinases (RTKs). Three additional members are included in this family (ErbB1/epidermal growth factor receptor (EGFR), ErbB3 and ErbB4), with which ErbB2 shares structural homology. The ErbB receptors are composed of an extracellular ligand-binding domain, a short hydrophobic transmembrane domain and cytosolic tail with intrinsic tyrosine kinase activity (only absent in ErbB3) [5]. By exhibiting a ligand-independent mode of activation and constituting the preferred heterodimerization partner of the remaining ErbB receptors, ErbB2 holds the most pronounced ability for potent and prolonged signal transduction. ErbB2 therefore carries an exponentially aggravated oncogenic and transforming potential and represents an excellent molecular candidate for selective anti-cancer targeted therapy [6,7]. In fact, the trastuzumab monoclonal antibody (MAb), targeting the subdomain IV of the ErbB2 extracellular region, has become the first personalized standard of care for patients harboring advanced ErbB2-positive GC, in combination with conventional chemotherapy [8]. However, the emergence of acquired tumor resistance, through still elusive molecular mechanisms, has limited the expected benefits from this therapeutic strategy [9,10].

Protein glycosylation is the enzymatic addition of sugar moieties to specific amino acid residues, and consists of a complex and finely regulated cellular process, representing the most abundant type of protein posttranslational modifications [11,12]. Glycosylation results from the well-orchestrated action of a wide range of enzymes and organelles and is acknowledged to be crucial in several cellular, physiological and pathological processes, including the carcinogenesis of multiple organs, such as the stomach [12,13]. The abnormal expression and activity of glycosyltransferase and glycosidase enzymes, as well as alterations in the structure and expression levels of cancer-associated glycan epitopes, have been consistently linked to the dismal prognosis of cancer patients (reviewed in [12]). Despite the remarkable advances made in glycan-based analytical techniques, many of the molecular mechanisms through which aberrant glycosylation actively tunes malignant cell behavior within the intricate context of tumor biology are still unknown [14].

RTK maturation is tightly regulated by protein N-linked glycosylation [15]. Furthermore, the glycosylation status of these transmembrane proteins has been shown to play a pivotal role in several aspects of receptor’s biology in the context of distinct human developmental and cancer models [16,17,18,19,20]. In particular, the extracellular domain of the ErbB2 receptor harbors seven putative sites for the anchorage of *N*-linked glycan structures (www.cbs.dtu.dk/NetNGlyc), five of which had their glycosylated status demonstrated in human mammary carcinoma cell lines [21,22]. However, protein glycosylation is highly context-dependent, which severely limits the extrapolation of relevant findings from one pathological setting to another [12]. In this sense, the detailed glycosylation profile of this oncogenic RTK within the context of GC requires further elucidation.

In the present study, we have performed a molecular characterization of four GC cell lines (NCI-N87, AGS, MKN45 and MKN74), including the transcription analysis of cancer-relevant glycosylation enzymes, as well as the expression and activation status of the ErbB family of receptors. Additional cellular and receptor-specific characterization of NCI-N87, an in vitro model of ErbB2 overexpression and hyperactivation, unveiled a highly heterogeneous and complex glycosylation pattern encompassing well-established tumor-associated glycan determinants, such as the endothelial selectin-ligand sialyl Lewis a (SLe^a^). The consistent expression of this glycan epitope and key enzymes of its biosynthetic pathway observed in ErbB2-overexpressing GC cells was further corroborated by in silico analysis. Moreover, an in silico association between the *ERBB2* and *FUT3* genes was disclosed in a cohort of GC patients. Finally, in vitro deglycosylation and antibody-mediated glycan-blocking of SLe^a^ drastically altered receptor’s expression and activation in ErbB2-overexpressing NCI-N87 GC cells. Altogether, these results portray the NCI-N87 cell line as a useful in vitro tool to study the regulatory role of glycans in ErbB2-driven gastric carcinogenesis.

## 2. Results

### 2.1. Expression and Activation of the Human Epidermal Growth Factor Receptor Family in Gastric Cancer Cell Lines

**Figure 1:**
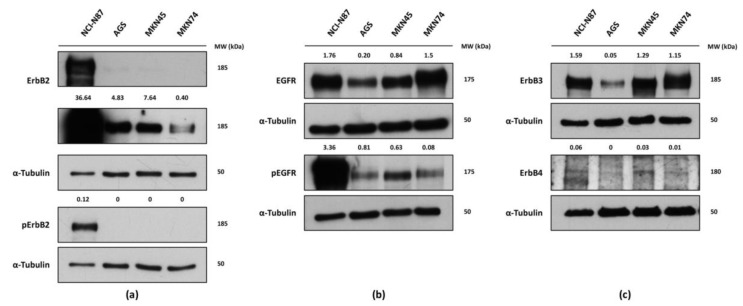
Western blot (WB) analysis of the total expression and activation (phosphorylated receptor) of the human epidermal growth factor receptor family members: (**a**) total and phosphorylated human epidermal growth factor receptor 2 (ErbB2 and pErbB2); (**b**) total and phosphorylated human epidermal growth factor receptor (EGFR and pEGFR); and (**c**) ErbB3 and ErbB4 in four gastric adenocarcinoma cell lines (NCI-N87, AGS, MKN45 and MKN74). Two distinct exposure times (a shorter and a longer one, from top to bottom, respectively) are depicted for the ErbB2 WB. α-Tubulin was used as a loading control. Band densities, normalized for the loading control (for total ErbB2 and EGFR) or both the loading control and total receptor (for pErbB2 and pEGFR), are depicted. All experiments were performed in triplicate and a representative WB analysis is depicted.

To characterize the expression pattern of the four RTKs integrating the ErbB family (ErbB1/EGFR, ErbB2, ErbB3 and ErbB4), Western blot (WB) analysis was performed on whole cell lysates from four GC cell lines (NCI-N87, AGS, MKN45 and MKN74), representative for the heterogeneous nature of gastric cancer regarding ErbB2 status (Figure 1). Furthermore, the phosphorylation status of the tyrosine-kinase domains of ErbB2 and EGFR receptors was additionally determined. In general, the four GC cell lines exhibited heterogeneous expression and activation of the analyzed RTKs. The *ERBB2*-amplified and well-differentiated NCI-N87 cell line displayed the highest levels of total ErbB2 protein, and was the only cell line with strong constitutive activation of this receptor (Figure 1a). ErbB2 expression was also detected in the remaining three GC cell lines, although in considerably lower levels. Despite similar levels of total EGFR expression being observed for NCI-N87 and MKN74, the former cell line displayed substantially higher receptor phosphorylation (Figure 1b). Although in substantially lower levels, endogenous EGFR activation was also detected in AGS, MKN45 and MKN74 cell lines. As for ErbB3, all cell lines displayed comparable protein levels of the receptor, with the exception of AGS where ErbB3 expression was lower (Figure 1c). Expression of ErbB4 was particularly low across the four screened GC cell lines.

### 2.2. Glycosyltransferase Expression in Gastric Cancer Cell Lines

**Figure 2:**
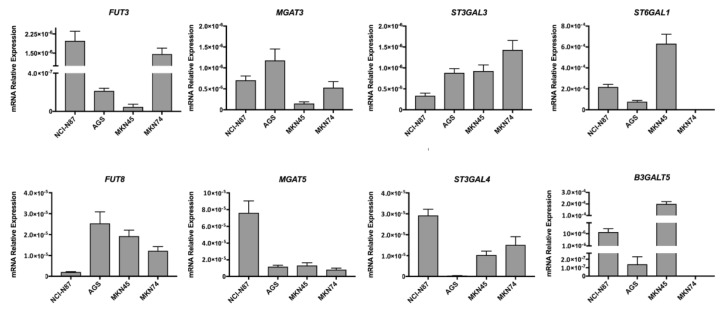
Real-time quantitative PCR (RT-qPCR) analysis of the relative transcript expression levels of eight cancer-relevant glycosyltransferase-coding genes (*FUT3*, *FUT8*, *MGAT3*, *MGAT5*, *ST3GAL3*, *ST3GAL4*, *ST6GAL1*, and *B3GALT5*) in four GC cell lines (NCI-N87, AGS, MKN45 and MKN74). For each cell line, the mRNA levels of a given gene are presented as 2^−∆Δ*C*t^. Target gene relative abundance was normalized to the mRNA levels of the *RNA18S5* endogenous control.

The presence of tumor-associated glycan epitopes at the surface of malignant cells is regulated by the coordinated expression and activity of specific glycosyltransferases. In this sense, prior to an ErbB2-targeted glycosylation analysis, we first evaluated the general glycophenotype of the four GC cell lines. The transcript levels of eight key genes encoding for glycosyltransferases previously reported to be implicated in human neoplastic transformation were assessed by real-time quantitative PCR (RT-qPCR) (Figure 2). The *FUT3* and *FUT8* genes encode for enzymes with fucosyltransferase activity responsible for the terminal (α1,3-linked) or core (α1,6-linked) fucosylation of their glycoconjugate targets, respectively. The *MGAT3* and *MGAT5* genes encode for enzymes that catalyze the addition of bisecting and branching *N*-acetylglucosamine (GlcNAc) during the biosynthesis of complex *N*-glycans, respectively. The *ST3GAL3* and *ST3GAL4* genes encode for sialyltransferases capable of adding α2,3-linked sialic acids to the terminal non-reducing end of glycan chains. The *ST6GAL1* gene encodes for an enzyme capable of transferring an α2,6-linked sialic acid monosaccharide to *N*-glycan substrates. Finally, The *B3GALT5* gene encodes for an enzyme involved in the biosynthesis of type 1 chains of oligosaccharides, which is prerequisite for the formation of Lewis a (Le^a^), Lewis b (Le^b^) and SLe^a^ antigens.

The obtained RT-qPCR uncovered a distinct glycosyltransferase expression signature for each of the screened GC cell lines. In particular, the *B3GALT5* gene showed an expression range of multiple orders of magnitude across the four screened cell lines. Indeed, the diffuse type MKN45 cell line exhibits remarkably higher levels of this enzyme’s mRNA when compared to the other cell lines, especially in the case of intestinal type MKN74 cells where no mRNA was detected. A similar expression pattern was observed for *ST6GAL1*. However, regarding *ST6GAL1*, a generally higher transcript expression was consistently observed across the four screened GC cell lines, with the exception of MKN74, in comparison to the remaining screened glyco-genes. Illustrative of the cell line-specific glycosyltransferase expression signature, the ErbB2-overexpressing NCI-N87 cell line displayed the highest expression of *FUT3*, *MGAT5* and *ST3GAL4* genes, but also the lowest detected transcript levels of both *FUT8* and *ST3GAL3*. The obtained results are in accordance with previous data published by our group, in respect to the GC cell lines and glycosyltransferase-coding genes commonly analyzed in both studies [23].

### 2.3. Glycosylation Profile of ErbB2-Overexpressing NCI-N87 Cells

**Figure 3:**
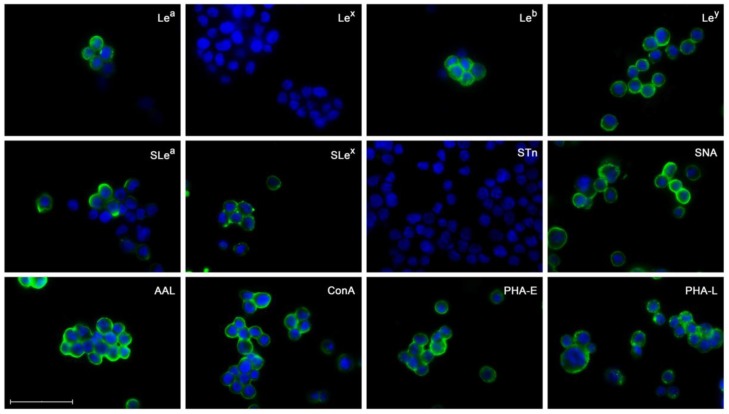
Immunofluorescent labeling of the glycan epitopes expressed at the cell membrane of the NCI-N87 cell line. The specifications of the used glycan-recognizing monoclonal antibodies (MAbs) (Lewis a (Le^a^), Lewis x (Le^x^), Lewis b (Le^b^), Lewis y (Le^y^), sialyl Lewis a (SLe^a^), sialyl Lewis x (SLe^x^) and sialyl Tn (STn)) and lectins (*Sambucus nigra* (SNA), *Aleuria aurantia* (AAL), Concanavalin A (ConA), *Phaseolus vulgaris* erythroagglutinin (PHA-E) and *Phaseolus vulgaris* leucoagglutinin (PHA-L)) are listed in Table 1. White scale bar indicates 50 μm. All experiments were performed in duplicate and a representative image for each antigen is depicted.

Since the NCI-N87 GC cell line exhibited the highest levels of both ErbB2 expression and activation, it was therefore selected for further glycan epitope characterization. Due to the subcellular localization of ErbB2, specific glycan-recognizing monoclonal antibodies and lectins were used for the immunofluorescent detection of the glycan structures expressed at the cell membrane of NCI-N87 cells (Figure 3). The obtained results unveiled a complex glycosylation signature, encompassing a range of structural and functionally diverse glycan structures, many of them previously described as key molecular players in the onset and progression of human cancer [12].

The cell membrane of NCI-N87 cells stained positive for all neutral (Le^a^, Le^b^ and Lewis y (Le^y^)) and sialylated (SLe^a^ and sialyl Lewis x (SLe^x^)) forms of the Lewis antigens, with the exception of Lewis x (Le^x^), for which no signal was detected. The expression of the tumor-associated sialyl Tn (STn) truncated *O*-glycan antigen was also undetected. This observation suggests that the detected terminal α2,6 sialic acid moieties by the lectin SNA are present on *N*-glycan chains. The presence at the cellular membrane of four additional cancer-relevant glycan structures (terminal and core fucose, high mannose, and bisecting and branching GlcNAc) was detected through their specific recognition by a panel of carbohydrate-binding lectins (*Aleuria aurantia* (AAL), Concanavalin A (ConA), *Phaseolus vulgaris* erythroagglutinin (PHA-E) and *Phaseolus vulgaris* leucoagglutinin (PHA-L), respectively). Regarding the enzymes responsible for the biosynthesis of distinct glycan determinants, these results are in agreement with the previously characterized transcription profile of this particular cell line (Figure 2). For example, the presence of SLe^x^, terminal α2,6 sialic acid, bisecting and branched complex *N*-glycan determinants, consistently match the detected expression of the *ST3GAL4*, *ST6GAL1*, *MGAT3* and *MGAT5* genes, respectively.

### 2.4. ErbB2 Glycan Signatures in NCI-N87 Cells

**Figure 4:**
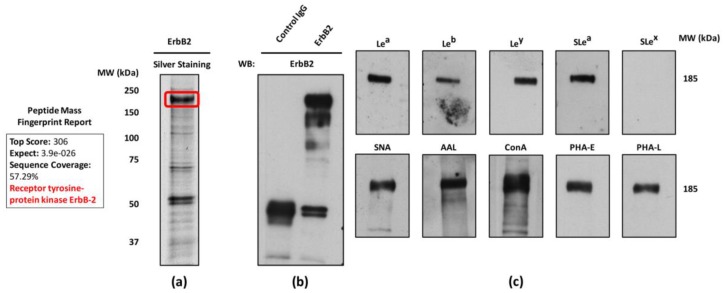
Glycan profiling of ErbB2 immunoprecipitated from NCI-N87 cells. (**a**) Silver staining of immunoprecipitated ErbB2 from NCI-N87 whole cell lysates and confirmation of receptor identity by matrix-assisted laser desorption ionization time-of-flight (MALDI/TOF-TOF) mass spectrometry. The excised and identified 185-kDa band is marked in red. The summary of the Peptide Mass Fingerprint analysis is also depicted; (**b**) ErbB2 WB for validation of the immunoprecipitation reaction. Normal rabbit IgGs were used as a control of the immunoprecipitation reaction; (**c**) WB and lectin blot analysis of ErbB2 glycan structures using glycan-recognizing MAbs and lectins (specifications are listed in Table 1). All WB experiments were performed in duplicate and a single representative analysis for each antigen and lectin is depicted.

With the aim of elucidating the ErbB2 glycosylation pattern in NCI-N87 cells, the receptor was immunoprecipitated from NCI-N87 total cell lysates. The yield and purity of receptor immunoprecipitation was evaluated by silver staining, and ErbB2 was validated by the subsequent peptide mass fingerprint analysis of the major 185-kDa band by matrix-assisted laser desorption ionization time-of-flight (MALDI/TOF-TOF) mass spectrometry (Figure 4a). The visible bands at 50 kDa correspond to the IgG heavy chains of the ErbB2-specific MAb used for receptor immunoprecipitation. As a control, a parallel immunoprecipitation reaction using normal rabbit IgGs was performed. As no signal in ErbB2 WB was detected, the specificity of receptor’s immunoprecipitation was validated (Figure 4b).

For the profiling of the glycan structures specifically carried by ErbB2 in NCI-N87 cells, the immunoprecipitated receptor was further characterized by both WB and lectin blot analysis with the previously used panel of glycan-recognizing antibodies and lectins found positive by immunofluorescence in NCI-N87 cells (Figure 4c). The 185-kDa band identified as the ErbB2 receptor displayed a specific glycosylation profile resembling the one of NCI-N87 cells. The only exception was SLe^x^, for which no signal was observed by WB. Indeed, when SLe^x^ WB was performed on NCI-N87 whole cell lysates, a single band was visible with a corresponding molecular weight higher than 185-kDa, indicating that the SLe^x^ epitope is carried by a protein other than ErbB2 in this particular GC cell line (data not shown). To our knowledge, this is the first study identifying ErbB2 as a carrier of the tumor-associated SLe^a^, a well-established molecular player in the malignant behavior of tumor cells.

### 2.5. Interplay Between ErbB2 and Sialyl Lewis a (SLe^a^) Expression

**Figure 5:**
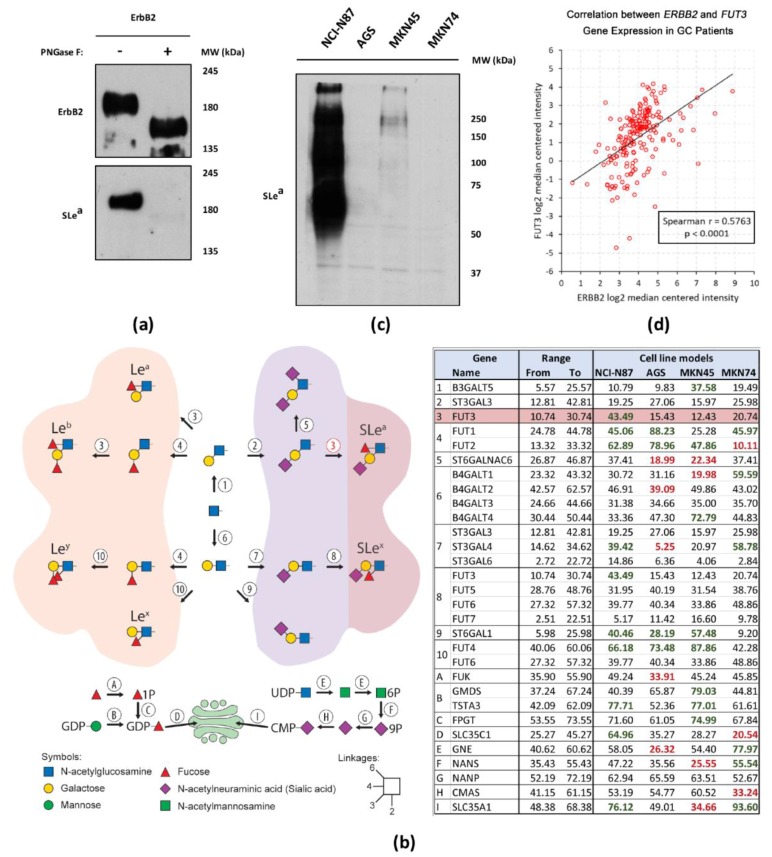
Association between ErbB2 expression and biosynthesis of *N*-linked SLe^a^ in GC cell lines. (**a**) WB of ErbB2 and SLe^a^ following receptor immunoprecipitation from NCI-N87 whole cell lysates and PNGase F digestion; (**b**) **Left** panel: Schematic representation of the glycosyltransferases (1–10) and enzymes responsible for the assembly of activated monosaccharides (A–I) integrating the biosynthetic pathways of Lewis antigens; **Right** panel: Relative transcriptomic abundance of the genes represented in the right panel across the four screened GC cell lines. The raw data on transcription levels were extracted from the Barretina CellLine data deposited in the Oncomine^TM^ database. Based on normalized transcription values of all 35 GC cell lines that were included in the dataset, a range of average expression values for GC cell lines was defined for each gene. Transcription values that were exceptionally high are highlighted in green and values that were exceptionally low in red; (**c**) WB of SLe^a^ expression in four GC cell lines. All experiments were performed in duplicate and a representative WB analysis is depicted; (**d**) Spearman’s rank correlation between the transcript levels of the *ERBB2* and *FUT3* genes in GC patients from the Ooi dataset available at the Oncomine^TM^ database.

Having identified ErbB2 as a protein carrier of the SLe^a^ antigen in ErbB2-overexpressing NCI-N87 cells, we aimed at dissecting the biosynthetic pathway of this cancer-relevant glycan epitope in this particular cell line, both in vitro and in silico. Lewis antigens constitute terminal structures in both *N*- and *O*-linked oligosaccharide chains [24]. To elucidate which type of SLe^a^-capped glycans are carried by ErbB2 in NCI-N87 cells, the immunoprecipitated receptor was digested with the PNGase F enzyme, a glycosidase capable of removing all *N*-linked glycans through the specific cleavage between the sugar’s innermost GlcNAc and the protein’s asparagine residue (Figure 5a). As expected, following PNGase F digestion, a clear shift in the molecular weight of ErbB2 was observed. This shift was accompanied by the disappearance of the 185-kDa SLe^a^ band, indicating that this terminal carbohydrate epitope is present on *N*-linked glycan chains.

To establish a valid causal association between the performed transcriptomic analysis of glycosyltransferase-coding genes and the expression of SLe^a^ in the NCI-N87 GC cell line (Figure 2 and Figure 3, respectively), in silico analysis, using the transcriptomic Barretina CellLine data deposited in the Oncomine^TM^ platform (www.oncomine.org), was performed. For this purpose, the either exceptionally high or low expression of each gene of interest, as compared to a total of 35 GC cell lines, was assessed in the four GC cell lines (Figure 5b, right panel). Amongst the four analyzed GC cell lines, NCI-N87 clearly exhibited the most robust expression profile of genes relevant for SLe^a^ biosynthesis and thus emerged as the most promising candidate to consistently express this glycan epitope. Besides the pronounced overexpression of SLe^a^-relevant genes (*FUT3*, *TSTA3*, *SLC35C1*, and *SLC35A1*), no downregulation of enzyme-coding genes directly implicated in SLe^a^ biosynthesis is observed, contrary to what occurs for the remaining three GC cell lines. The generated in silico results are in robust alignment with the ones obtained in the performed transcriptomic analysis (Figure 2). Illustrating this observation is the overexpression of *FUT3* by the *ERBB2*-amplified NCI-N87, and of *B3GALT5* by MKN45. It is also worth noting that, although unrelated to SLe^a^ biosynthesis, *ST3GAL4* is downregulated in AGS cells, and *ST6GAL1* is downregulated in MKN45, NCI-N87 and AGS when compared to MKN74 cell line. To validate the robustness of the in silico data, the four GC cell lines were screened for the expression of SLe^a^ by WB (Figure 5c). Remarkably, and in agreement with the in silico results, the only GC cell line exhibiting a strong SLe^a^ immunodetection was NCI-N87. Our data therefore suggest that the marked upregulation of SLe^a^ in NCI-N87 cells may be driven by high *FUT3* expression levels.

Finally, we sought to investigate whether the apparent ErbB2-SLe^a^ crosstalk could also be observed in patients harboring GC. For this purpose, an in silico analysis based on the transcriptomic data of the Ooi dataset extracted from the Oncomine^TM^ platform was carried out. Among the 200 primary gastric carcinomas of the referred dataset, a statistically highly significant positive association (Spearman’s rank correlation *p* < 0.0001) between the expression of *ERBB2* and *FUT3* genes was observed (Figure 5d). The latter gene encodes for the FucT-III glycosyltransferase, also known as the Lewis enzyme, which is solely responsible for the biosynthesis of SLe^a^ [25]. In conclusion, a higher *ERBB2* expression was associated to *FUT3* upregulation in the GC patients, and vice-versa.

### 2.6. Inhibition of Glycosylation and Glycan Epitope-Blocking Disrupt ErbB2 Expression and Activation

**Figure 6:**
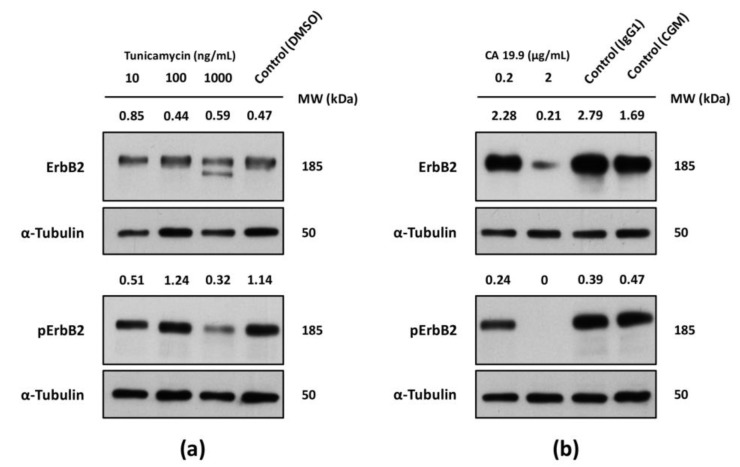
WB analysis of the total and phosphorylated ErbB2 following in vitro deglycosylation or MAb-mediated blockage of the SLe^a^ epitope in the NCI-N87 cell line. Cells were treated for 24 h with different concentrations of: (**a**) tunicamycin in complete growth medium; and (**b**) CA 19.9 MAb diluted in simple growth medium with the corresponding controls. α-Tubulin was used as a loading control. Band densities, normalized for the loading control (for total ErbB2) or both the loading control and total receptor (for pErbB2), are depicted. All experiments were performed in duplicate and a representative WB analysis is depicted. CGM: complete growth medium.

Protein *N*-linked glycosylation actively regulates RTK maturation, translocation to the cell membrane and signaling activity [26]. Having identified the major types of glycans carried by ErbB2 in NCI-N87 cells, we aimed at assessing the functional impact of glycosylation-targeting agents on receptor’s expression and activation status in this particular cell line. NCI-N87 cells were subjected to increasing concentrations of two distinct treatment regimens: tunicamycin, a natural inhibitor of the enzyme catalyzing the first step of *N*-glycan biosynthesis, and the CA 19.9 MAb targeting the SLe^a^ epitope. Following 24 h of treatment, WB analysis of both total and phosphorylated forms of ErbB2 was performed on freshly collected protein extracts (Figure 6). Only the highest concentration of the tunicamycin compound (1000 ng/mL) exerted a pronounced effect on the levels of both total and activated protein (Figure 6a). In regard to total receptor’s expression, a second band with lower molecular weight strongly indicates the presence of a deglycosylated version of ErbB2, in addition to the heavier 185-kDa band corresponding to the fully glycosylated receptor. Moreover, the same concentration of tunicamycin led to a significant reduction of receptor’s phosphorylation. Since ErbB2 phosphorylation seems to be only detected at 185-kDa, the observed reduction can be due to a general decrease in the abundance of the fully matured transmembrane receptor, which is the one targeted by phosphorylation. As for the treatment of NCI-N87 cells with the CA 19.9 MAb, drastic effects on both forms of the receptor could also be clearly observed. While the lowest CA 19.9 concentration (0.2 µg/mL) produced no visible effects on ErbB2 total expression, and a mild, yet detectable, reduction on the ErbB2 phosphorylation, the highest MAb concentration (2 µg/mL) led to a severe reduction of total receptor’s levels and a complete abrogation of receptor’s phosphorylation.

## 3. Discussion

The extracellular region of the ErbB2 mitogenic receptor is a well-documented target for extensive glycosylation, which has been shown to actively promote human carcinogenesis through the regulation of receptor’s total expression, subcellular localization and signaling potential [15,26,27]. However, the ErbB2 glycosylation signature in GC cells and the molecular mechanisms through which it may tune receptor biology towards malignancy remain poorly understood. The present study aimed at disclosing glycan determinants carried by ErbB2 that may act as functional regulators of the receptor in ErbB2-driven gastric carcinogenesis. The NCI-N87 cell line was therefore selected, amongst the four screened cell lines, as the most appropriate in vitro model of ErbB2-positive GC.

Despite the similar high levels of total EGFR expression observed in NCI-N87 and MKN74, the former GC cell line exhibited the most pronounced activation of this receptor, and also of ErbB2, as previously described [28]. This observation may reflect the homo- or hetero-oligomerization capacity of the ErbB transmembrane receptors, which constitutes a well-established pre-requisite for intracellular activation [29]. The ErbB2 protein is overexpressed in NCI-N87 cells as a direct consequence of gene amplification and constitutes the preferred heterodimerization partner of EGFR [30,31]. Therefore, in the NCI-N87 GC cell line, the high levels of expression of the ErbB2 RTK at the cell membrane lead not only to its own hyperactivation, but also of the remaining members of the family. This is in agreement with the significantly lower levels of both EGFR endogenous activation ErbB2 total expression observed in the three other GC cell lines. However, whether ErbB2-specific glycosylation plays an active part on dimer formation, although reported for other ErbB receptors, remains a question to be addressed [16,32,33,34,35].

The elaborate shaping of the cellular glycan landscape depends on the highly controlled and dynamic crosstalk between both glycosyltransferases and glycosidases [36]. Interestingly, the observed expression levels of the selected glycogenes agree with the known competing enzymatic role that these glycosyltransferases play in the cell, in response to both external and internal stimuli. For example, the *MGAT3* gene displays a generally lower expression across all four GC cell lines compared to *MGAT5*. The same can be observed for *ST3GAL3* and *ST3GAL4*, respectively.

Although the expression of specific glycan epitopes depends on multiple factors, such as enzyme activity, substrate recognition and availability of both donor and acceptor molecule, in NCI-N87 cells we have observed a remarkable match between the transcriptomic profile of cancer-relevant glycosyltransferase-coding genes and the expression of their respective glycan products. This is illustrated by the correspondence between the observed expression of the *MGAT3*, *MGAT5*, *ST3GAL3* and *ST3GAL4*, and *ST6GAL1* genes, and the immunodetection of bisected and branched *N*-glycans, sialylated versions of the Lewis antigens, and the terminally α2,6-sialylated *N*-glycan chains, respectively, both at the cellular and receptor levels. Some of the disclosed glycan signatures have been consistently reported as active promoters of human gastric carcinogenesis. The expression of the Le^b^ antigen at the surface of the stomach’s epithelial lining is crucial for *Helicobacter pylori* (*H. pylori*) pathogenic colonization of the host’s gastric mucosa [37]. The α2,3 sialylated versions of the Lewis antigens, SLe^x^ and SLe^a^, constitute additional motifs for *H. pylori* adhesion. Moreover, they are known ligands of selectins expressed at the surface of endothelial cells, and therefore function as molecular docking sites during the metastatic dissemination of circulating tumor cells (reviewed in [38]).

The highest mRNA expression of *MGAT5* was detected in ErbB2-overexpressing NCI-N87 cells. Interestingly, a previous study reported that the transcriptional activation of the *MGAT5* gene and the activity of its translated product, the GnT-V enzyme, were directly regulated by the ErbB2 oncogenic receptor and key transducers of its downstream signaling pathway [39,40]. Furthermore, in another study, the overexpression of the *MGAT5* gene in mammary cells potentiated ErbB2-mediated malignancy in mouse models [41]. These observations suggest that branched *N*-glycans, the direct products of the *MGAT5* gene, and here disclosed as part of ErbB2 glycosylation signature, may function as active regulators of ErbB2-mediated signaling in NCI-N87 cells.

This study identified ErbB2 as a previously unreported protein carrier of the tumor-associated SLe^a^ epitope. In addition, both in silico and in vitro analysis of genes involved in SLe^a^ biosynthesis corroborate this finding, as illustrated by *FUT3* overexpression in NCI-N87 cells. The *FUT3* gene encodes for the only enzyme capable of the in vivo biosynthesis of SLe^a^ [25]. In this sense, the disclosed statistically significant positive correlation between *ERBB2* and *FUT3* expression in GC patients additionally strengthens this interplay. Moreover, the dramatic impact observed on both total and phosphorylated ErbB2, following treatment of the NCI-N87 cells with an anti-SLe^a^-specific MAb, further supports the association between ErbB2 status and SLe^a^ expression in this particular cell line. These results are in agreement with a previous study, where treatment of ErbB2-overexpressing tumor cells with anti-Le^y^ MAb produced a similar impact in the receptor’s expression [27]. The decreased detection of both total and activated ErbB2 following treatment with CA 19.9 MAb may reflect a rapid receptor internalization and recycling from the cell membrane following antibody-binding [42]. The tunicamycin treatment, on the other hand, by completely abrogating the *N*-glycosylation pathway, prevents the “naked” and misfolded receptor from accumulating at the cell membrane and, therefore, of becoming activated [15]. Our findings have uncovered an interesting regulatory interplay that should be further elucidated. Additional studies using the administration of other glycosylation inhibitors, glycan specific antibodies or glycosidases are warranted and may further unravel the molecular basis of the underlying mechanism.

Although a steady decline of its incidence and mortality rates has been observed over the past decades, GC still remains a lethal disease with worldwide coverage [43]. Furthermore, the discovery of novel and reliable biomarkers for early disease diagnosis remains a challenging issue in GC research. On the other hand, the abnormal expression of specific glycoconjugates by malignant cells benefits from a degree of unprecedented specificity and constitutes, in this way, a foreseeable promising source of novel diagnostic, prognostic and therapeutic molecular tools. The SLe^a^ carbohydrate antigen is deeply involved in multiple molecular processes underlying neoplastic transformation [44,45,46,47,48]. Moreover, the frequent upregulation of circulating SLe^a^-modified glycoconjugates in individuals bearing gastrointestinal tumors led to the clinical implementation of the CA 19.9 serological assay. The SLe^a^-specific CA 19.9 assay has become widely used for monitoring disease progression and patient response to therapy [49,50,51]. Since the shedding of the ErbB2 extracellular domain by malignant cells into the bloodstream is commonly observed in patients harboring ErbB2-positive cancers, circulating ErbB2 has been previously proposed as a novel circulating biomarker and, curiously, had its performance found comparable to the one of CA 19.9 [52,53,54]. In this light, our findings open a new research line: if proven that ErbB2 is, in fact, an in vivo protein carrier of SLe^a^ in ErbB2-positive GC patients, assessing ErbB2 modified with SLe^a^ in the sera of patients might surpass the clinical performance of either circulating ErbB2 or SLe^a^.

Regardless of the initial enthusiasm following drug development, the use of the trastuzumab MAb in the treatment of ErbB2-expressing advanced GC frequently fails to ameliorate the overall survival of the majority of patients [55]. Nevertheless, due to its consistently observed overexpression in a significant fraction of patients, the ErbB2 transmembrane receptor remains one of the most promising molecular targets for the clinical management of GC. In the near future, it is likely that advances in the field of glycobiology will allow the improvement of classic protein targets in human malignancy, alongside the widespread usage of novel glycoconjugates as effective theranostic tools. In this study, the NCI-N87 cell line is reported as an interesting in vitro model system to study the regulatory role of glycans in ErbB2-positive GC.

## 4. Materials and Methods

### 4.1. Gastric Cancer Cell Lines and Cell Culture

Four human GC cell lines (NCI-N87, AGS, MKN45, and MKN74) were used in this study. The intestinal type NCI-N87, AGS and MKN74 cell lines were obtained from the American Type Culture Collection (Manassas, VA, USA) and the diffuse type MKN45 cell line was obtained from the Japanese Cancer Research Bank (Tsukuba, Japan). Cell line identity was authenticated by standard short tandem-repeat based DNA profiling. All four cell lines were grown in a monolayer culture and maintained at 37 °C in an atmosphere of 5% CO_2_, in complete growth medium: RPMI 1640 GlutaMAX (Gibco, Thermo Fisher Scientific, Waltham, MA, USA) supplemented with 10% heat-inactivated fetal bovine serum (Biowest, Riverside, MO, USA). Cultured cell lines were routinely tested for mycoplasma contamination.

### 4.2. Western Blotting

Total protein lysates were collected by scrapping confluent cultures of each cell line using lysis buffer 17 (R&D Systems, Minneapolis, MN, USA) supplemented with 1 mM sodium orthovanadate (Sigma-Aldrich, St. Louis, MO, USA), 1 mM phenylmethanesulfonyl fluoride (PMSF) (Sigma-Aldrich) and cOmplete^TM^ protease inhibitor cocktail (Roche, Basel, Switzerland; Sigma-Aldrich). Protein concentration of whole cell lysates was determined using the DC protein assay (BioRad, Hercules, CA, USA). Distinct amounts of total protein lysates (15 µg for ErbB2, pErbB2, EGFR and pEGFR, 30 µg for ErbB3, 50 µg for ErbB4, and 50 µg for SLe^a^ analysis) were denaturated and negatively charged by boiling for 10 min in Laemmli buffer containing 5% β-mercaptoethanol at 100 °C, subjected to 8% SDS-PAGE, and blotted onto a nitrocellulose membrane (GE Healthcare Life Sciences, Chicago, IL, USA). Following 1 h blocking at room temperature with either 5% non-fat milk or 5% of bovine serum albumin (BSA) (Sigma-Aldrich) in 0.1% Tween^®^ 20 (Sigma-Aldrich) in tris buffered saline (TBS), membranes were incubated overnight at 4 °C with primary antibodies. For biotinylated lectins, membranes were blocked with 2% polyvinylpyrrolidone (Sigma-Aldrich) in TBS overnight at 4 °C and, after washing, incubated for 1 h at room temperature with the corresponding lectin. Then, membranes were washed three times for 10 min with TBS-Tween^®^ 20, and were incubated for 1 h at room temperature with peroxidase-conjugated secondary antibodies D1/2000 (Santa Cruz Biotechnology, Dallas, TX, USA, and Jackson ImmunoResearch, West Grove, PA, USA) or peroxidase-conjugated streptavidin D1/100,000 (GE Healthcare) for Western and lectin blot, respectively. Following three additional washes in TBS-Tween^®^ 20, chemiluminescence signal was obtained using the ECL WB detection reagent and films (both from GE Healthcare). All WB experiments were performed in triplicate. The information regarding dilutions used for antibodies and lectins is provided in Table 1.

### 4.3. Quantitative Real-Time PCR

Total RNA from the four GC cell lines was isolated using TRI reagent (Sigma-Aldrich, St. Louis, MO, USA), according to manufacturer’s instructions. Following spectrophotometric assessment of RNA yield and quality, 5 µg of each cell line’s total RNA were reverse-transcribed into single-stranded cDNA using Superscript^TM^ II Reverse Transcriptase kit (Invitrogen, Thermo Fisher Scientific), following manufacturer’s recommendations. Glycosyltransferase mRNA expression levels were quantified by RT-qPCR using TaqMan^TM^ Universal PCR Master Mix II, no UNG, and the specific TaqMan^TM^ gene expression assays, listed below, in a 7500 Fast Real-Time PCR System (Applied Biosystems, Foster City, CA, USA; Thermo Fisher Scientific). The mRNA expression levels of 18S Ribosomal 5 (*RNA18S5*) endogenous control were also measured for normalization of target gene abundance. RT-qPCR was carried out in triplicate for each biological sample and in duplicate for the negative controls. The following TaqMan^TM^ gene expression assays were used: *FUT3* (Hs01868572_s1), *FUT8* (Hs00189535_m1), *MGAT3* (Hs02379589_s1), *MGAT5* (Hs01073268_m1), *ST3GAL3* (Hs00544035_m1), *ST3GAL4* (Hs00920871_m1), *ST6GAL1* (Hs00949382_m1), *B3GALT5* (Hs00707757_s1), and *RNA18S5* (Hs99999901_s1). Data were analyzed by ΔΔ*C*_t_ method [60].

### 4.4. Immunofluorescence Labeling of Glycan Epitopes

Following trypsinization, NCI-N87 cells were washed twice with ice-cold phosphate buffered saline (PBS, pH 7.4) and air-dried overnight on 12-well microscope glass slides (Marienfeld Superior, Lauda-Königshofen, Germany). Prior to immunofluorescence, cells were fixed for 5 min at room temperature with 4% paraformaldehyde (Alfa Aesar, Haverhill, MA, USA; Thermo Fisher Scientific). Single immunofluorescence was performed as follows. Samples were rinsed three times in PBS for 5 min and permeabilized with 0.5% Triton^TM^ X-100 (Sigma-Aldrich) in PBS for 10 min at 4 °C. Samples were rinsed three times with PBS and incubated for 30 min with goat non-immune serum (Dako, Agilent, Santa Clara, CA, USA) D1/5 in PBS 10% BSA or PBS 10% BSA, for MAb and lectin immunofluorescent labeling, respectively. Samples were rinsed in PBS and incubated overnight at 4 °C with MAbs and biotinylated lectins (Table 1) diluted in PBS containing 5% BSA (MAbs) or PBS (lectins). Samples were washed three times for 5 min in PBS and incubated for 45 min at room temperature with secondary antibodies labeled with either Alexa Fluor^®^ 488 or 594 fluorochromes (Thermo Fisher Scientific) or FITC-conjugated streptavidin, for MAb- and lectin-mediated immunolabeling, respectively. Samples were washed three times for 5 min in PBS, incubated with 1 µg/mL DAPI for 5 min at room temperature for nuclear staining and mounted in VectaShield (Vector Laboratories, Burlingame, CA, USA). Images were acquired with a Zeiss Axio cam MRm and the AxioVision Rel. 4.8 software (both from Zeiss, Oberkochen, Germany). All experiments were performed in duplicate.

### 4.5. ErbB2 Immunoprecipitation

For ErbB2 immunoprecipitation, 400 µg of total protein from NCI-N87 whole cell lysates were pre-cleared with 30 µL of Protein G Sepharose Fast Flow beads (GE Healthcare Life Sciences) for 2 h at 4 °C. Separately, 60 µL of Protein G Sepharose Fast Flow beads were conjugated with 5 µL of the 29D8 ErbB2 MAb (Cell Signaling Technology, Danvers, MA, USA) for 2 h at 4 °C. Immunoprecipitation was performed by incubation of the pre-cleared lysate with the antibody-conjugated beads overnight at 4 °C. All steps were performed with gentle agitation. In parallel, the same immunoprecipitation was performed using normal rabbit IgGs (Merck Millipore, Burlington, MA, USA) as a negative control. After washing with ice-cold 0.1% Triton X-100 in PBS, the immune complexes were released and denaturated by boiling for 10 min in Laemmli buffer containing 5% β-mercaptoethanol at 100 °C. The immunoprecipitates were subjected to SDS-PAGE followed by either silver staining, using Pierce^®^ Silver Stain for Mass Spectrometry kit (Thermo Fisher Scientific) according to the manufacturer’s instructions, or WB as described in Section 4.2. All experiments were performed in duplicate.

### 4.6. ErbB2 Identification by Matrix-Assisted Laser Desorption Ionization Time-Of-Flight (MALDI/TOF-TOF) Mass Spectrometry

Following silver staining, the major visible band was excised from the gel, washed with 50% acetonitrile in 50 mM ammonium bicarbonate, digested for 3 h with 20 ng of trypsin at 37 °C, and analyzed on a MALDI mass spectrometer (4800 Plus MALDI TOF/TOF Analyzer, SCIEX) as described in [61]. Proteins were identified by Peptide Mass Fingerprint using the Mascot software v2.5.1 (Matrix Science, London, UK). Protein searches were performed against the UniProt protein sequence database for the *Homo sapiens* taxonomic selection (2017_08, UP000005640 reviewed proteome, canonical proteins). The established search parameters were: up two missed cleavages allowed, cysteine carbamidomethylation as a fixed modification and methionine oxidation as a variable modification. The peptide tolerance was 20 ppm. Protein scores greater than 56 were considered to be significant (*p* < 0.05).

### 4.7. ErbB2 Digestion with PNGase F

Following immunoprecipitation, bead-conjugated immune complexes were denaturated for 10 min at 100 °C with denaturating buffer, and digested with 375 units of PNGase F (both from New England Biolabs, Ipswich, MA, USA) overnight at 37 °C. The immunoprecipitated deglycosylated proteins were subjected to 8% SDS-PAGE together with the corresponding control sample (parallel reaction without PNGase F) and subsequent WB was performed as described in Section 4.2. All experiments were performed in duplicate.

### 4.8. Tunicamycin and Cancer Antigen 19.9 (CA 19.9) MAb Treatment

Following trypsinization of confluent NCI-N87 cultures, 5 × 10^5^ cells were seeded in 6-well plates and allowed to attach for 24 h. Cells were then incubated for another 24 h with 0.01, 0.1 and 1 µg/mL of tunicamycin (Sigma-Aldrich) and 0.2 and 2 µg/mL of CA 19.9 MAb (Santa Cruz Biotechnology), in RPMI 1640 GlutaMAX supplemented with 10% heat-inactivated fetal bovine serum and simple RPMI 1640 GlutaMAX, respectively. DMSO (0.05%) and 2 µg/mL of mouse IgG1 isotype control (R&D Systems) were used as controls for tunicamycin and CA 19.9 treatment, respectively. Regarding CA 19.9 treatment, an additional control of complete growth medium was included. Finally, total protein extracts were collected for each experimental condition and WB was performed as described in Section 4.2. All experiments were performed in duplicate.

### 4.9. Bioinformatic and Statistical Analysis

The log2 median centered gene expression data of human cancer cell lines and gastric cancer patients were extracted from the Oncomine^TM^ platform (www.oncomine.org) [62].

The gene expression profile analysis of the cell lines NCI-N87, AGS, MKN45 and MKN74 was performed using the Barretina CellLine dataset [63]. In this dataset, the gene expression values of 913 human cancer cell lines were analyzed by Affymetrix GeneChip Human Genome U133 Plus 2.0 Array. After extracting the gene expression data, we normalized the log2 median centered intensity levels (i) of each gene probe among all 913 human cancer cell lines of the dataset into values ranging from 0 to 100 by using the formula:

normalized(i)=(value(i)−minimum valuemaximum value−minimum value)×100

The median for each normalized gene probe was determined among the 35 human gastric cancer cell lines. We defined the normal expression range for each gene transcript as the median ±10. 

When more than one probe for a gene of interest was present, we chose the best probe according to following criteria: (1) probe accession _at over _a_at over _x_at over _s_at; (2) highest probe grading as annotated in www.affymetrix.com; and (3) highest sequence identity between probe and target sequence. If more than one probe remained after applying these criteria, the average among the remaining probes was used.

The association analysis was performed in GraphPad Prism 7 using the Ooi Gastric dataset [64]. The Ooi Gastric dataset comprises expression data of 200 malignant primary gastric tumors obtained by Affymetrix GeneChip Human Genome U133 Plus 2.0 Array. Both datasets, Barretina and Ooi, used the same criteria to select the probes for *ERBB2* and *FUT3* as described above. The *ERBB2* and *FUT3* data were not normally distributed (determined by Shapiro–Wilk normality test) and association was therefore analyzed using Spearman’s correlation analysis.

## Figures and Tables

**Table 1:** Specificity and working conditions of the antibodies and lectins used for Western blot (WB) and immunofluorescence (IF) analysis.

Antibody Clone/Lectin	Antigen	Working Dilution		Supplier
		IF	WB	
29D8	ErbB2	-	1:1000	Cell Signaling Technology
Polyclonal	pErbB2 (Tyr1221/1222)	-	1:1000	Cell Signaling Technology
D38B1	EGFR (ErbB1)	-	1:1000	Cell Signaling Technology
D7A5	pEGFR (Tyr1068)	-	1:1000	Cell Signaling Technology
D22C5	ErbB3	-	1:2000	Cell Signaling Technology
111B2	ErbB4	-	1:1000	Cell Signaling Technology
DM1A	α-Tubulin	-	1:10,000	Sigma-Aldrich
CA 19.9 (241)	SLe^a^	1:500	1:1000	Santa Cruz Biotechnology
SPM279	Le^a^	-	1:200	Santa Cruz Biotechnology
CSLEX1	SLe^x^	1:80	1:1000	BD Pharmingen^TM^
B72.3	STn	1:5	-	[56]
CA3F4	Le^a^	1:5	-	[57]
BG6 (T218)	Le^b^	1:50	1:200	Signet
SH1	Le^x^	1:5	Undiluted	[58]
AH6	Le^y^	1:2	Undiluted	[59]
*Aleuria aurantia* lectin (AAL)	Fucα6GlcNAc	1:500	1:3000	Vector Labs
*Phaseolus vulgaris* leucoagglutinin (PHA-L)	Galβ4GlcNAcβ6 (GlcNAcβ2Manα3) Manα3	1:500	1:2000	Vector Labs
*Phaseolus vulgaris* erythroagglutinin (PHA-E)	Galβ4GlcNAcβ2Manα6 (GlcNAcβ4) (GlcNAcβ4Manα3) Manβ4	1:500	1:2000	Vector Labs
Concanavalin A (Con A)	αMan, αGlc	1:500	1:2000	Vector Labs
*Sambucus nigra* lectin (SNA)	Neu5Acα6Gal/GalNAc	1:500	1:3000	Vector Labs

## Abbreviations

BSA	Bovine serum albumin
CA 19.9	Cancer antigen 19.9
EGFR	Epidermal growth factor receptor
ErbB2	Human epidermal growth factor receptor 2
GC	Gastric cancer
GlcNAc	*N*-Acetylglucosamine
Le^a^	Lewis a
Le^b^	Lewis b
Le^x^	Lewis x
Le^y^	Lewis y
MAb	Monoclonal antibody
PBS	Phosphate buffered saline
RT-qPCR	Real-time quantitative PCR
RTKs	Receptor tyrosine kinases
SLe^a^	Sialyl Lewis a
SLe^x^	Sialyl Lewis x
STn	Sialyl Tn
TBS	Tris buffered saline
WB	Western blot

## References

[1] Ferlay J., Steliarova-Foucher E., Lortet-Tieulent J., Rosso S., Coebergh J.W., Comber H., Forman D., Bray F. (2013). Cancer Incidence and Mortality Patterns in Europe: Estimates for 40 Countries in 2012. Eur. J. Cancer.

[2] Waddell T., Verheij M., Allum W., Cunningham D., Cervantes A., Arnold D. (2013). Gastric Cancer: Esmo–Esso–Estro Clinical Practice Guidelines for Diagnosis, Treatment and Follow-Up. Ann. Oncol..

[3] Yan M., Schwaederle M., Arguello D., Millis S.Z., Gatalica Z., Kurzrock R. (2015). HER2 Expression Status in Diverse Cancers: Review of Results from 37,992 Patients. Cancer Metastasis Rev..

[4] Gravalos C., Jimeno A. (2008). HER2 in Gastric Cancer: A New Prognostic Factor and a Novel Therapeutic Target. Ann. Oncol..

[5] Burgess A.W., Cho H.S., Eigenbrot C., Ferguson K.M., Garrett T.P., Leahy D.J., Lemmon M.A., Sliwkowski M.X., Ward C.W., Yokoyama S. (2003). An Open-and-Shut Case? Recent Insights into the Activation of EGF/ErbB Receptors. Mol. Cell.

[6] Hynes N.E., Stern D.F. (1994). The Biology of Erbb-2/Nue/Her-2 and Its Role in Cancer. Biochim. Biophys. Acta.

[7] Hynes N.E., Lane H.A. (2005). ERBB Receptors and Cancer: The Complexity of Targeted Inhibitors. Nat. Rev. Cancer.

[8] Bang Y.J., van Cutsem E., Feyereislova A., Chung H.C., Shen L., Sawaki A., Lordick F., Ohtsu A., Omuro Y., Satoh T. (2010). Trastuzumab in Combination with Chemotherapy Versus Chemotherapy Alone for Treatment of Her2-Positive Advanced Gastric or Gastro-Oesophageal Junction Cancer (Toga): A Phase 3, Open-Label, Randomised Controlled Trial. Lancet.

[9] Roukos D.H. (2010). Targeting Gastric Cancer with Trastuzumab: New Clinical Practice and Innovative Developments to Overcome Resistance. Ann. Surg. Oncol..

[10] Shah M.A., Xu R.H., Bang Y.J., Hoff P.M., Liu T., Herráez-Baranda L.A., Xia F., Garg A., Shing M., Tabernero J. (2017). Heloise: Phase Iiib Randomized Multicenter Study Comparing Standard-of-Care and Higher-Dose Trastuzumab Regimens Combined with Chemotherapy as First-Line Therapy in Patients with Human Epidermal Growth Factor Receptor 2-Positive Metastatic Gastric or Gastroesophageal Junction Adenocarcinoma. J. Clin. Oncol..

[11] Varki A., Stanley P., Schachter H., Taniguchi N. (2009). Essentials of Glycobiology.

[12] Pinho S.S., Reis C.A. (2015). Glycosylation in Cancer: Mechanisms and Clinical Implications. Nat. Rev. Cancer.

[13] Pinho S.S., Carvalho S., Marcos-Pinto R., Magalhães A., Oliveira C., Gu J., Dinis-Ribeiro M., Carneiro F., Seruca R., Reis C.A. (2013). Gastric Cancer: Adding Glycosylation to the Equation. Trends Mol. Med..

[14] Mereiter S., Balmaña M., Gomes J., Magalhães A., Reis C.A. (2016). Glycomic Approaches for the Discovery of Targets in Gastrointestinal Cancer. Front. Oncol..

[15] Contessa J.N., Bhojani M.S., Freeze H.H., Rehemtulla A., Lawrence T.S. (2008). Inhibition of N-Linked Glycosylation Disrupts Receptor Tyrosine Kinase Signaling in Tumor Cells. Cancer Res..

[16] Liu Y.C., Yen H.Y., Chen C.Y., Chen C.H., Cheng P.F., Juan Y.H., Chen C.H., Khoo K.H., Yu C.J., Yang P.C. (2011). Sialylation and Fucosylation of Epidermal Growth Factor Receptor Suppress Its Dimerization and Activation in Lung Cancer Cells. Proc. Natl. Acad. Sci. USA.

[17] Matsumoto K., Yokote H., Arao T., Maegawa M., Tanaka K., Fujita Y., Shimizu C., Hanafusa T., Fujiwara Y., Nishio K. (2008). N-Glycan Fucosylation of Epidermal Growth Factor Receptor Modulates Receptor Activity and Sensitivity to Epidermal Growth Factor Receptor Tyrosine Kinase Inhibitor. Cancer Sci..

[18] Wang X., Gu J., Ihara H., Miyoshi E., Honke K., Taniguchi N. (2006). Core Fucosylation Regulates Epidermal Growth Factor Receptor-Mediated Intracellular Signaling. J. Biol. Chem..

[19] Lopez-Sambrooks C., Shrimal S., Khodier C., Flaherty D.P., Rinis N., Charest J.C., Gao N., Zhao P., Wells L., Lewis T.A. (2016). Oligosaccharyltransferase Inhibition Induces Senescence in Rtk-Driven Tumor Cells. Nat. Chem. Biol..

[20] Mereiter S., Magalhães A., Adamczyk B., Jin C., Almeida A., Drici L., Ibáñez-Vea M., Gomes C., Ferreira J.A., Afonso L.P. (2016). Glycomic Analysis of Gastric Carcinoma Cells Discloses Glycans as Modulators of Ron Receptor Tyrosine Kinase Activation in Cancer. Biochim. Biophys. Acta.

[21] Frei A.P., Jeon O.Y., Kilcher S., Moest H., Henning L.M., Jost C., Plückthun A., Mercer J., Aebersold R., Carreira E.M. (2012). Direct Identification of Ligand-Receptor Interactions on Living Cells and Tissues. Nat. Biotechnol..

[22] Watanabe M., Terasawa K., Kaneshiro K., Uchimura H., Yamamoto R., Fukuyama Y., Shimizu K., Sato T.A., Tanaka K. (2013). Improvement of Mass Spectrometry Analysis of Glycoproteins by Maldi-Ms Using 3-Aminoquinoline/Α-Cyano-4-Hydroxycinnamic Acid. Anal. Bioanal. Chem..

[23] Carvalho A.S., Harduin-Lepers A., Magalhaes A., Machado E., Mendes N., Costa L.T., Matthiesen R., Almeida R., Costa J., Reis C.A. (2010). Differential Expression of Α-2, 3-Sialyltransferases and Α-1, 3/4-Fucosyltransferases Regulates the Levels of Sialyl Lewis a and Sialyl Lewis X in Gastrointestinal Carcinoma Cells. Int. J. Biochem. Cell Biol..

[24] Sterner E., Flanagan N., Gildersleeve J.C. (2016). Perspectives on Anti-Glycan Antibodies Gleaned from Development of a Community Resource Database. ACS Chem. Biol..

[25] Kudo T., Narimatsu H. (2014). Fucosyltransferase 3. Gdp-Fucose Lactosamine Α1, 3/4-Fucosyltransferase. Lea and Leb Histo-Blood Groups (Fut3, Lewis Enzyme). Handbook of Glycosyltransferases and Related Genes.

[26] Ohtsubo K., Marth J.D. (2006). Glycosylation in Cellular Mechanisms of Health and Disease. Cell.

[27] Klinger M., Farhan H., Just H., Drobny H., Himmler G., Loibner H., Mudde G.C., Freissmuth M., Sexl V. (2004). Antibodies Directed against Lewis-Y Antigen Inhibit Signaling of Lewis-Y Modified Erbb Receptors. Cancer Res..

[28] Tanner M., Hollmén M., Junttila T.T., Kapanen A.I., Tommola S., Soini Y., Helin H., Salo J., Joensuu H., Sihvo E. (2005). Amplification of HER-2 in Gastric Carcinoma: Association with Topoisomerase IIα Gene Amplification, Intestinal Type, Poor Prognosis and Sensitivity to Trastuzumab. Ann. Oncol..

[29] Brennan P.J., Kumagai T., Berezov A., Murali R., Greene M.I. (2000). HER2/Neu: Mechanisms of Dimerization/Oligomerization. Oncogene.

[30] Graus-Porta D., Beerli R.R., Hynes N.E. (1995). Single-Chain Antibody-Mediated Intracellular Retention of Erbb-2 Impairs Neu Differentiation Factor and Epidermal Growth Factor Signaling. Mol. Cell. Biol..

[31] Tzahar E., Waterman H., Chen X., Levkowitz G., Karunagaran D., Lavi S., Ratzkin B.J., Yarden Y. (1996). A Hierarchical Network of Interreceptor Interactions Determines Signal Transduction by Neu Differentiation Factor/Neuregulin and Epidermal Growth Factor. Mol. Cell. Biol..

[32] Tsuda T., Ikeda Y., Taniguchi N. (2000). The Asn-420-Linked Sugar Chain in Human Epidermal Growth Factor Receptor Suppresses Ligand-Independent Spontaneous Oligomerization Possible Role of a Specific Sugar Chain in Controllable Receptor Activation. J. Biol. Chem..

[33] Fernandes H., Cohen S., Bishayee S. (2001). Glycosylation-Induced Conformational Modification Positively Regulates Receptor-Receptor Association a Study with an Aberrant Epidermal Growth Factor Receptor (Egfrviii/Δegfr) Expressed in Cancer Cells. J. Biol. Chem..

[34] Yokoe S., Takahashi M., Asahi M., Lee S.H., Li W., Osumi D., Miyoshi E., Taniguchi N. (2007). The Asn418-Linked N-Glycan of Erbb3 Plays a Crucial Role in Preventing Spontaneous Heterodimerization and Tumor Promotion. Cancer Res..

[35] Takahashi M., Yokoe S., Asahi M., Lee S.H., Li W., Osumi D., Miyoshi E., Taniguchi N. (2008). N-Glycan of Erbb Family Plays a Crucial Role in Dimer Formation and Tumor Promotion. Biochim. Biophys. Acta.

[36] Ashkani J., Naidoo K.J. (2016). Glycosyltransferase Gene Expression Profiles Classify Cancer Types and Propose Prognostic Subtypes. Sci. Rep..

[37] Magalhães A., Rossez Y., Robbe-Masselot C., Maes E., Gomes J., Shevtsova A., Bugaytsova J., Borén T., Reis C.A. (2016). Muc5ac Gastric Mucin Glycosylation Is Shaped by Fut2 Activity and Functionally Impacts Helicobacter Pylori Binding. Sci. Rep..

[38] Trinchera M., Aronica A., Dall’Olio F. (2017). Selectin Ligands Sialyl-Lewis a and Sialyl-Lewis X in Gastrointestinal Cancers. Biology.

[39] Chen L., Zhang W., Fregien N., Pierce M. (1998). The Her-2/Neu Oncogene Stimulates the Transcription of N-Acetylglucosaminyltransferase V and Expression of Its Cell Surface Oligosaccharide Products. Oncogene.

[40] Buckhaults P., Chen L., Fregien N., Pierce M. (1997). Transcriptional Regulation of *N*-acetylglucosaminyltransferase V by the Srconcogene. J. Biol. Chem..

[41] Guo H.B., Johnson H., Randolph M., Nagy T., Blalock R., Pierce M. (2010). Specific Posttranslational Modification Regulates Early Events in Mammary Carcinoma Formation. Proc. Natl Acad. Sci. USA.

[42] Hadari Y.R., Doody J.F., Wang Y., Patel S.N., Apblett R.L., Loizos N., Pereira D.S., Witte L., Bohlen P., Hicklin D.J. The Igg1 Monoclonal Antibody Cetuximab Induces Degradation of the Epidermal Growth Factor Receptor. Proceedings of the ASCO Gastrointestinal Cancers Symposium.

[43] Anderson W.F., Camargo M.C., Fraumeni J.F., Correa P., Rosenberg P.S., Rabkin C.S. (2010). Age-Specific Trends in Incidence of Noncardia Gastric Cancer in US Adults. JAMA.

[44] Weston B.W., Hiller K.M., Mayben J.P., Manousos G.A., Bendt K.M., Liu R., Cusack J.C. (1999). Expression of Human Α (1, 3) Fucosyltransferase Antisense Sequences Inhibits Selectin-Mediated Adhesion and Liver Metastasis of Colon Carcinoma Cells. Cancer Res..

[45] Opolski A., Laskowska A., Madej J., Wietrzyk J., Kłopocki A., Radzikowski C., Ugorski M. (1998). Metastatic Potential of Human Cx-1 Colon Adenocarcinoma Cells is Dependent on the Expression of Sialosyl Le a Antigen. Clin. Exp. Metastasis.

[46] Mahdavi J., Sondén B., Hurtig M., Olfat F.O., Forsberg L., Roche N., Angstrom J., Larsson T., Teneberg S., Karlsson K.A. (2002). Helicobacter Pylori Saba Adhesin in Persistent Infection and Chronic Inflammation. Science.

[47] Hiller K.M., Mayben J.P., Bendt K.M., Manousos G.A., Senger K., Cameron H.S., Weston B.W. (2000). Transfection of Α (1, 3) Fucosyltransferase Antisense Sequences Impairs the Proliferative and Tumorigenic Ability of Human Colon Carcinoma Cells. Mol. Carcinog..

[48] Terraneo L., Avagliano L., Caretti A., Bianciardi P., Tosi D., Bulfamante G.P., Samaja M., Trinchera M. (2013). Expression of Carbohydrate-Antigen Sialyl-Lewis a on Colon Cancer Cells Promotes Xenograft Growth and Angiogenesis in Nude Mice. Int. J. Biochem. Cell Biol..

[49] Steinberg W. (1990). The Clinical Utility of the Ca 19–9 Tumor-Associated Antigen. Am. J. Gastroenterol..

[50] Holdenrieder S., Pagliaro L., Morgenstern D., Dayyani F. (2016). Clinically Meaningful Use of Blood Tumor Markers in Oncology. BioMed Res. Int..

[51] Reis C.A., Osorio H., Silva L., Gomes C., David L. (2010). Alterations in Glycosylation as Biomarkers for Cancer Detection. J. Clin. Pathol..

[52] Perng C.L., Lin H.J., Lee S.D. (1994). Serum C-Erbb-2 Oncoprotein in the Diagnosis of Gastric Cancer in Comparison with Ca 19–9, Cea, Tpa, Ca 125 and Afp. Zhonghua yi xue za zhi.

[53] Molina R., Jo J., Filella X., Bruix J., Castells A., Hague M., Ballesta A.M. (1997). Serum Levels of C-Erbb-2 (Her-2/Neu) in Patients with Malignant and Non-Malignant Diseases. Tumor Biol..

[54] Kono K., Naganuma H., Sekikawa T., Amemiya H., Takahashi A., Iizuka H., Matsumoto Y. (2000). Serum Level of Her-2/Neu in Patients with Gastric Cancer: Correlation with Her-2/Neu Overexpression in Gastric Carcinoma Tissue. Tumor Biol..

[55] Wong H., Yau T. (2013). Molecular Targeted Therapies in Advanced Gastric Cancer: Does Tumor Histology Matter?. Therap. Adv. Gastroenterol..

[56] Colcher D., Hand P.H., Nuti M., Schlom J. (1981). A Spectrum of Monoclonal Antibodies Reactive with Human Mammary Tumor Cells. Proc. Natl. Acad. Sci. USA.

[57] Young W.W., Portoukalian J., Hakomori S. (1981). Two Monoclonal Anticarbohydrate Antibodies Directed to Glycosphingolipids with a Lacto-*N*-Glycosyl Type II Chain. J. Biol. Chem..

[58] Fukushi Y., Hakomori S., Nudelman E., Cochran N. (1984). Novel Fucolipids Accumulating in Human Adenocarcinoma. II. Selective Isolation of Hybridoma Antibodies That Differentially Recognize Mono-, Di-, and Trifucosylated Type 2 Chain. J. Biol. Chem..

[59] Abe K., McKibbin J.M., Hakomori S. (1983). The Monoclonal Antibody Directed to Difucosylated Type 2 Chain (Fuc Alpha 1 Leads to 2gal Beta 1 Leads to 4 [Fuc Alpha 1 Leads to 3] Glcnac; Y Determinant). J. Biol. Chem..

[60] Livak K.J., Schmittgen T.D. (2001). Analysis of Relative Gene Expression Data Using Real-Time Quantitative Pcr and the 2-Δδct Method. Methods.

[61] Ferreirinha P., Correia A., Teixeira-Coelho M., Osório H., Teixeira L., Rocha A., Vilanova M. (2016). Mucosal Immunization Confers Long-Term Protection against Intragastrically Established Neospora Caninum Infection. Vaccine.

[62] Rhodes D.R., Yu J., Shanker K., Deshpande N., Varambally R., Ghosh D., Barrette T., Pandey A., Chinnaiyan A.M. (2004). Oncomine: A Cancer Microarray Database and Integrated Data-Mining Platform. Neoplasia.

[63] Barretina J., Caponigro G., Stransky N., Venkatesan K., Margolin A.A., Kim S., Wilson C.J., Lehár J., Kryukov G.V., Sonkin D. (2012). The Cancer Cell Line Encyclopedia Enables Predictive Modelling of Anticancer Drug Sensitivity. Nature.

[64] Ooi W.F., Xing M., Xu C., Yao X., Ramlee M.K., Lim M.C., Cao F., Lim K., Babu D., Poon L.F. (2016). Epigenomic Profiling of Primary Gastric Adenocarcinoma Reveals Super-Enhancer Heterogeneity. Nat. Commun..

